# Stromal Expression of Heat-Shock Protein 27 Is Associated with Worse Clinical Outcome in Patients with Colorectal Cancer Lung Metastases

**DOI:** 10.1371/journal.pone.0120724

**Published:** 2015-03-20

**Authors:** Thomas Schweiger, Christoph Nikolowsky, Patrick Starlinger, Denise Traxler, Matthias Zimmermann, Peter Birner, Balazs Hegedüs, Balazs Dome, Michael Bergmann, Michael Mildner, Walter Klepetko, Konrad Hoetzenecker, Hendrik Jan Ankersmit

**Affiliations:** 1 Department of Thoracic Surgery, Medical University of Vienna, Vienna, Austria; 2 Christian Doppler Laboratory for Cardiac and Thoracic Diagnosis and Regeneration, Medical University of Vienna, Vienna, Austria; 3 Department of General Surgery, Medical University of Vienna, Vienna, Austria; 4 Clinical Institute of Pathology, Medical University of Vienna, Vienna, Austria; 5 2nd Department of Pathology, Semmelweis University, Budapest, Hungary; 6 Molecular Oncology Research Group, Hungarian Academy of Sciences-Semmelweis University, Budapest, Hungary; 7 Comprehensive Cancer Center, Medical University of Vienna, Vienna, Austria; 8 National Koranyi Institute of Pulmonology, Budapest, Hungary; 9 Department of Thoracic Surgery, National Institute of Oncology-Semmelweis University, Budapest, Hungary; 10 Department of Biomedical Imaging and Image-guided Therapy, Division of Molecular and Gender Imaging, Medical University of Vienna, Vienna, Austria; 11 Department of Dermatology, Medical University of Vienna, Vienna, Austria; Technische Universitaet Muenchen, GERMANY

## Abstract

**Background:**

Pulmonary metastases are common in patients with primary colorectal cancer (CRC). Heat-shock protein 27 (Hsp27) is upregulated in activated fibroblasts during wound healing and systemically elevated in various diseases. Cancer-associated fibroblasts (CAFs) are also thought to play a role as prognostic and predictive markers in various malignancies including CRC. Surprisingly, the expression of Hsp27 has never been assessed in CAFs. Therefore we aimed to investigate the expression level of Hsp27 in CAFs and its clinical implications in patients with CRC lung metastases.

**Methods:**

FFPE tissue samples from 51 pulmonary metastases (PMs) and 33 paired primary tumors were evaluated for alpha-SMA, CD31, Hsp27 and vimentin expression by immunohistochemistry and correlated with clinicopathological variables. 25 liver metastases served as control group. Moreover, serum samples (n=10) before and after pulmonary metastasectomy were assessed for circulating phospho-Hsp27 and total Hsp27 by ELISA.

**Results:**

Stromal expression of Hsp27 was observed in all PM and showed strong correlation with alpha-SMA (*P*<0.001) and vimentin (*P*<0.001). Strong stromal Hsp27 was associated with higher microvessel density in primary CRC and PM. Moreover, high stromal Hsp27 and αSMA expression were associated with decreased recurrence-free survival after pulmonary metastasectomy (*P*=0.018 and *P*=0.008, respectively) and overall survival (*P*=0.031 and *P*=0.017, respectively). Serum levels of phospho- and total Hsp27 dropped after metastasectomy to levels comparable to healthy controls.

**Conclusions:**

Herein we describe for the first time that Hsp27 is highly expressed in tumor stroma of CRC. Stromal α-SMA and Hsp27 expressions correlate with the clinical outcome after pulmonary metastasectomy. Moreover, serum Hsp27 might pose a future marker for metastatic disease in CRC.

## Introduction

Colorectal cancer (CRC) is, after lung cancer, the second most common cause of cancer-related death in Europe [1]. More than one fifth of the patients with CRC present with metastases already at time of diagnosis of the primary cancer and the same proportion will develop metastases during the course of disease [1, 2]. The lungs are the second most common site of distant metastasis, making pulmonary metastases (PM) an essential contributor to the high mortality of CRC.

Besides cancer cells themselves, a tumor comprises stromal cells. The interactions of stromal and cancer cells is thought to be a major determinant of the tumor behavior and response to therapy [3, 4]. Cellular components of the stroma are fibroblasts, endothelial cells, immune cells and pericytes [5]. Cancer-associated fibroblasts (CAF), and especially activated fibroblasts, play a major role in the tumor-stroma network, similar to dermal fibroblasts in wound healing. This contributed to the description of tumors as “wounds that do not heal” by Dvorak *et al*. in the late 80’s [6]. CAF contribute to various tumor-promoting characteristics like extra-cellular matrix turnover, tumor growth, angiogenesis and metastasis [7, 8]. Due to the expression of α-smooth muscle actin (α-SMA), activated CAF are often described as myofibroblasts. They have also been shown to be positive for fibroblast-activation protein-α/seprase, palladin and vimentin [9–12]. Recently, efforts have been made to characterize the “signature” of these fibroblasts by proteome and gene expression profiling [13, 14].

In the context of benign diseases, wound healing and keloid formation it is well known that activated fibroblasts express high levels of heat-shock protein 27 (Hsp27), which is crucial for fibroblast adhesion, contractility and motility [15–17]. The TGF-beta induced p38-MAPK pathway is the key regulator in the induction of Hsp27 in smooth muscle cells and myofibroblasts [18, 19]. Once synthesized, two main functions of Hsp27 are critical in wound healing process: promoting myofibroblast motility and angiogenesis. Hsp27 is involved in the stabilization of actin filaments and of SNAIL, an inducer of epithelial-mesenchymal transition. Both mechanisms contribute to the induction of the myofibroblastic phenotype [20, 21]. Another function of Hsp27 executed in a paracrine manner is enhancing angiogenesis. It was demonstrated that extracellular Hsp27 leads to NfκB activation and subsequent expression of the proangiogenic factors VEGF and interleukin-8 (IL-8) in endothelial cells [22]. Together with others, our group could show that an overexpression of Hsp27 is strongly linked to several benign and malign pathologies of the lung associated with fibroblast activation, including emphysema/chronic obstructive pulmonary disease (COPD), idiopathic pulmonary fibrosis and non-small cell lung cancer [21, 23–26].

Given the fact that activated fibroblasts play an important role in the progression of malignant disease as well as in various non-malignant diseases of the lung, we aimed to investigate the prevalence of Hsp27 positive tumor stroma in CRC lung metastases and corresponding primary tumors. Furthermore, we sought to describe the implications on the clinical outcome after metastasectomy and the presence of cellular and secreted Hsp27 in these patients.

## Materials and Methods

### Study population

From April 2009 to November 2013, all consecutive cases of pulmonary metastasectomy from primary CRC and appropriate tissue samples were included in this study. Patients received diagnostic work-up including thoracic and abdominal computed tomography (CT). If patients had undergone pulmonary metastasectomy before, specimen of the first metastasectomy was also examined and the date of the first metastasectomy was used for outcome calculation. Lung metastasis free survival (LMFS) was defined as the time between diagnosis of the primary tumor and diagnosis of the metastatic spreading to the lung. R0 resection was achieved in all patients. Of 33 patients, specimens of the corresponding primary tumor could be obtained. Follow-up examinations were carried out in 3 to 6 months intervals. Recurrence-free survival (RFS) was defined as the period from the first pulmonary metastasectomy to evidence of recurrent disease at any site verified by CT scans. In 10 consecutive cases, serum samples obtained before metastasectomy and during follow-up were available. Follow-up serum samples were collected 3 to 6 months after surgery. Additionally, serum samples from age-, gender- and smoking status- matched healthy volunteers were collected. All patients gave their written informed consent prior to blood collection and participation. A study cohort of 25 consecutive patients with resected CRC liver metastases served as additional control group. The study was approved by the ethics committee of the Medical University of Vienna (EK 91/2006, EK1194/2011 and EK1044/2012) and was conducted according to the declaration of Helsinki. The current study cohort is based on previous published works by our group.[27, 28]

### Immunohistochemistry and immunofluorescence

Immunohistochemical staining was performed according to a standard protocol. Shortly, formalin-fixed, paraffin-embedded tissue specimens were cut in 4-μm thick sections and deparaffinized. Heat-mediated antigen retrieval was performed. Endogenous peroxidase activity was quenched with 0.3% hydrogen peroxide. Sections were incubated with the appropriate primary antibody for 1h at room temperature. For immunohistochemistry, as secondary step, the polymer- based ImmPRESS kit (Vector Laboratories, Burlingame, California) was used according to the manufacturer’s protocol. 3,3’-Diaminobenzidine (DAB) was used as substrate (Vector Laboratories, Burlingame, California). Finally, the sections were counterstained with hematoxylin. Vimentin staining was performed in a Ventana ES Immunostainer System (Ventana Medical Systems Inc., Tucson, AZ, USA). For immunofluorescence, appropriate fluorescent secondary antibodies were used and cell nuclei were counterstained with DAPI (Sigma Aldrich, St. Louis, MO, USA). As negative controls, the primary antibody was omitted. Positive controls were tissue samples with known presence of the respective target protein. A detailed list of antibodies and dilutions is provided as supplementary information (S1 Table).

#### Scoring of stained tumor cells

Immunohistochemistry staining score (IHC score) was calculated as described previously [29]. The percentage of positive tumor cells could reach values between 0 and 100%, and were multiplied by the staining intensity (0 to 3). Thus, IHC scores could range from 0 to 300. Two blinded observers rated the staining. In case that the two ratings differed, the slide was discussed and re-evaluated. The continuous IHC score was dichotomized by applying the median score of metastases or primary tumors as cut-off value.

#### Scoring of stained stromal cells

The sections were scored semiquantitatively as described previously [10]. Briefly, the slides were screened at low magnification and evaluated for their staining intensity in the stromal cells in the tumor center. The stromal staining was assessed as grade 0 (negative, <1% positive stromal cells), grade 1+ (low,1–10% positive stromal cells), grade 2+ (intermediate, >10–50% positive stromal cells) and grade 3+ (strong, >50% positive stromal cells) by two blinded observers. In case that the two ratings differed, the slide was discussed, re-evaluated and a consensus was reached on all slides.

#### Determination of microvessel density

Microvessel density (MVD) was measured using the “hotspot” method, as published elsewhere [30]. In brief, the slides were screened at low magnification to identify the area with the greatest number of CD31-positive microvessels (“hotspot”). MVD was determined by counting all microvessels at 200x magnification (corresponding to 0.95 mm^2^). Mean values were calculated from two independently counted densities. In case of strong inter-observer discrepancy, the slide was reevaluated.

### Enzyme-linked immunosorbent assay (ELISA)

Serum samples were assessed by commercially available human Hsp27, phospho-Hsp27 and interleukin-8 ELISA kits (all R&D Systems, Minneapolis, USA). Measurement was conducted according to the manufacturer’s instructions. The samples were assayed in duplicates. The absorbance was measured at 450nm using a plate reader (PerkinElmer, Waltham, USA), compared to a standard curve with known protein content and converted to pg/mL.

### Statistical analysis

All obtained data was evaluated statistically using SPSS 19 (SPSS Inc., Chicago, USA) and GraphPad Prism 6 (GraphPad Software Inc., California, USA). Student’s t-test was used to compare means of two independent groups, paired t-test for dependent groups and expressed as mean±standard deviation (SD). Kaplan-Meier curves and log-rank test were used to compare survival functions. Chi-square test and Fisher’s exact test were used to compare binominal variables. All tests were two-sided. P-values equal or below 0.05 were considered as statistically significant.

## Results

Tissue specimens of PM from a total of 51 consecutive patients were available. Median age at metastasectomy was 63 years (range 33–83). 29 (56.9.1%) male and 22 (43.1%) female patients were included. The clinicopathological characteristics of the included patients are summarized in Table 1.

**Table 1:** Descriptive data on patient, primary tumor and metastases characteristics stratified by stromal Hsp27 and α-SMA expression (N = 51).

				Stromal Hsp27 expression in PM							Stromal alpha-SMA expression in PM						
		Total		low		intermediate		high		X^2^	low		intermediate		high		X^2^
		N = 51	%	N = 17	%	N = 17	%	N = 17	%	p-value	N = 14	%	N = 23	%	N = 14	%	p-value
**Patients**																	
**Sex**	**Male**	29	56.9	10	19.6	8	15.7	11	21.6	0.571	8	15.7	12	23.5	9	17.6	0.771
	**Female**	22	43.1	7	13.7	9	17.6	6	11.8		6	11.8	11	21.6	5	9.8	
**Age (years)**	**Median**	63		66		62		63		0.618^a^	67		62		61.5		0.383^a^
	**Range**	33–83		47–83		37–78		33–74			50–83		37–78		33–74		
**Primary tumor**																	
**Location**	**Colon**	27	52.9	9	17.6	8	15.7	10	19.6	0.790	6	11.8	14	27.5	7	13.7	0.549
	**Rectum**	24	47.1	8	15.7	9	17.6	7	13.7		8	15.7	9	17.6	7	13.7	
**T stage**	**pT1**	1	2.1	1	2.1	0	0.0	0	0.0		1	2.1	0	0.0	0	0.0	
	**pT2**	7	14.6	1	2.1	3	6.3	3	6.3	0.109^b^	1	2.1	3	6.3	3	6.3	0.032^b^
	**pT3**	34	70.8	11	22.9	11	22.9	12	25.0		8	16.7	16	33.3	10	20.8	
	**pT4**	6	12.5	2	4.2	3	6.3	1	2.1		3	6.3	3	6.3	0	0.0	
	**N/A**	3	-														
**N stage**	**pN0**	21	43.8	9	18.8	6	12.5	6	12.5	0.169^b^	7	14.6	9	18.8	5	10.4	0.299^b^
	**pN1**	11	22.9	2	4.2	6	12.5	3	6.3		2	4.2	7	14.6	2	4.2	
	**pN2**	16	33.3	4	8.3	5	10.4	7	14.6		4	8.3	6	12.5	6	12.5	
	**N/A**	3	-														
**Grading**	**G1**	2	3.9	1	2.0	1	2.0	0	0.0		1	2.0	1	2.0	0	0.0	
	**G2**	42	82.4	13	25.5	14	27.5	15	29.4	0.487^b^	11	21.6	19	37.3	12	23.5	0.713^b^
	**G3**	7	13.7	3	5.9	2	3.9	2	3.9		2	3.9	3	5.9	2	3.9	
**Stromal HSP27**	**low**	6	18.2	0	0.0	2	6.1	4	12.1		0	0.0	3	9.1	3	9.1	
	**intermediate**	15	45.5	6	18.2	6	18.2	3	9.1	0.053^b^	5	15.2	8	24.2	2	6.1	0.081^b^
	**high**	12	36.4	4	12.1	4	12.1	4	12.1		4	12.1	5	15.2	3	9.1	
	**N/A**	18	-														
**Stromal alpha-SMA**	**low**	8	24.2	2	6.1	3	9.1	3	9.1		3	9.1	3	9.1	2	6.1	
	**intermediate**	14	42.4	4	12.1	6	18.2	4	12.1	0.711^b^	3	9.1	7	21.1	4	12.1	0.779^b^
	**high**	11	33.3	4	12.1	3	9.1	4	12.1		3	9.1	6	18.2	2	6.1	
	**N/A**	18															
**Pulmonary metastasis**																	
**Microvessel density**	**Mean**	39.0		33.0		40.9		43.0		0.169^e^	32.4		39.5		44.6		0.136^e^
	**Range**	12–96		12–56		16–76		19–96			12–76		18–65		19–96		
**Previous liver metastasis**	**Yes**	16	31.4	5	9.8	6	11.8	5	9.8	0.913	1	2.0	12	23.5	3	5.9	0.004^b^
	**No**	35	68.6	12	23.5	11	21.6	12	23.5		13	25.5	11	21.6	11	21.6	
**No. of nodules**	**1**	33	64.7	12	23.5	9	17.6	12	23.5	0.462	8	15.7	14	27.5	11	21.6	0.236^b^
	**>1**	18	35.3	5	9.8	8	15.7	5	9.8		6	27.5	9	45.1	3	27.5	

^a^Kruskal-Wallis test;

^b^Fisher’s exact test;

^e^Oneway-ANOVA; LMFS: Lung-metastasis free survival after primary tumor

### Hsp27 is highly expressed in cancer-associated stroma of lung metastases

**Fig 1:**
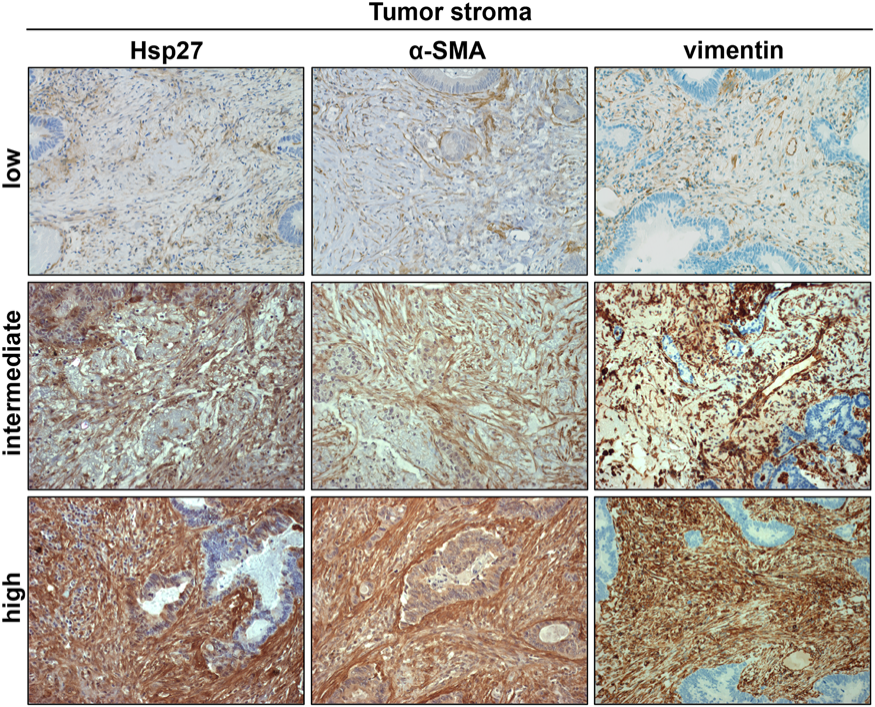
Representative images showing pulmonary metastases with low, intermediate and high intensity of positive tumor stroma stained for Hsp27 and α-SMA. Stromal fibroblasts were further identified by vimentin staining. (DAB substrate, same tumor specimen per row, 200x magnification).

To assess the expression level of Hsp27 in cancer-associated stroma, tissue sections were stained for Hsp27 and vimentin (Fig. 1). The Hsp27 expression of tumor stroma in PM was scored in 0 (0%), 17 (33%), 17 (33%), 17 (33%) cases as 0, 1+, 2+ and 3+, respectively. The correlation of the stromal Hsp27 staining with patient and tumor characteristics is depicted in Table 1. Staining intensity of the tumor cells was determined separately (S1A Fig). 23 (45%) metastases were scored as Hsp27 highly positive and 28 (55%) metastases as low/negative (IHC score range 0–160; median/cut off 30). There was no correlation between tumor and stromal Hsp27 expression in pulmonary metastases (Chi square test; *P* = 0.237). 33 corresponding primary tumors were available. The stromal Hsp27 expression in the primary tumors 0 (0%), 6 (18%), 15 (46%) and 12 (36%) were scored as 0, 1+, 2+ and 3+, respectively. Determining the Hsp27 staining in the tumor cells in the primary tumor tissue, 14 (42%) of the cases were scored as Hsp27 positive and 19 (58%) as low/negative (IHC score range 5–100; median/cut off 70).

### Hsp27 is co-expressed with α-SMA and vimentin in the stroma of PMs

**Fig 2:**
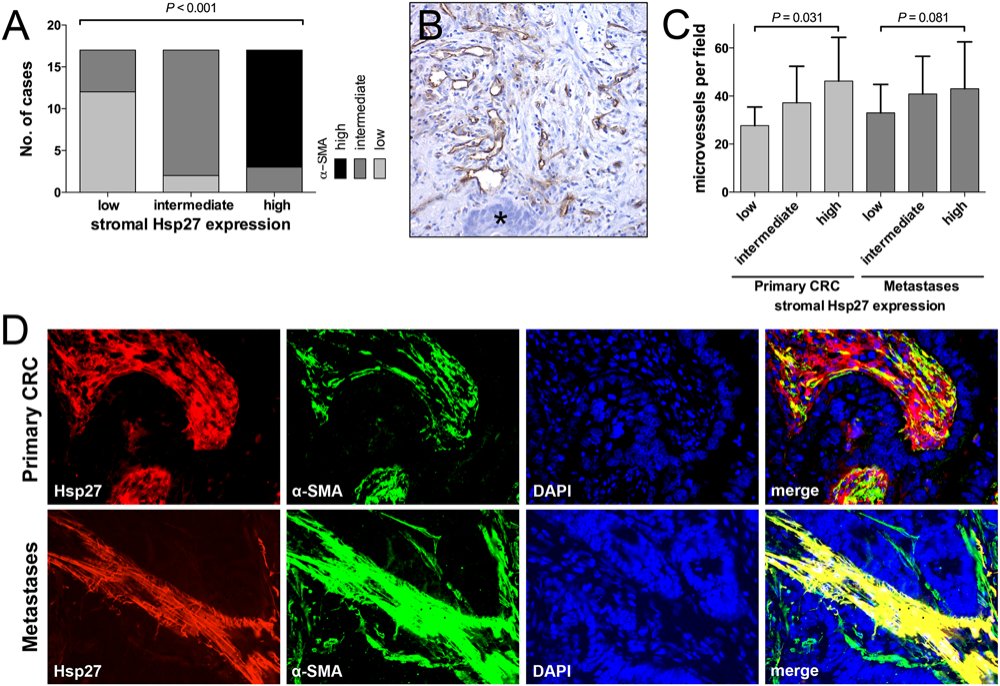
The degree of Hsp27 expression score in the tumor stroma correlated significantly with the expression of stromal α-SMA (A). CD31-positive microvessels surrounded by tumor stroma next to tumor cells (asterisk) (B). MVD was significantly increased in primary tumors and metastases with strong stromal Hsp27 expression (C). Immunofluorescence showed a co-expression of stromal Hsp27 and α-SMA especially in PM (400x magnification) (D).

Activated tumor stroma has been described as highly α-SMA positive in primary and metastatic CRC [12, 31]. Therefore, the specimens were stained for α-SMA. The expression of α-SMA in the stroma was rated in the same semiquantitative manner as the Hsp27 staining. 0 (0%), 13 (25.5%), 24 (51%) and 13 (25.5%) cases were scored as 0, 1+, 2+ and 3+, respectively. The staining score distribution according to the clinicopathological variables is depicted in Table 1. Expression levels of α-SMA correlated significantly with Hsp27 expression in PMs (*P* < 0.001, Fig. 2A). This was also observed in liver metastases (S2 Fig). To further assess the stromal co-expression of α-SMA and Hsp27, representative slides of primary and metastatic tumors were co-labeled for α-SMA and Hsp27 by immunofluorescence. A strong co-expression could be observed especially in PMs (Fig. 2D). Tumor stroma with strong Hsp27 and α-SMA staining was also highly positive for vimentin (S3 Table).

### MVD is increased in tissue samples with Hsp27-positive tumor stroma

Recently it was shown that Hsp27 mediates angiogenesis [22]. We therefore determined the MVD by CD31 staining and correlated it with the Hsp27 expressions (Fig. 2B and 2C). In PM, we found a trend towards higher MVD in samples with high Hsp27 levels. MVD was 33.0±2.9, 40.1±3.8 and 43.0±4.7 for 1+, 2+ and 3+ Hsp27 intensity (mean±SD). However, this trend did not reach the level of significance (*P =* 0.081). Similarly, in primary CRC with high stroma levels of Hsp27, significantly more microvessels could be found (27.7±3.2, 37.2±4.0 and 46.4±5.2 for 1+, 2+ and 3+ Hsp27 intensity, respectively; *P =* 0.031). A detailed description of MVD stratified by clinicopathological characteristics is provided in S2 Table.

### Stromal Hsp27 is associated with poor outcome after pulmonary metastasectomy

**Fig 3:**
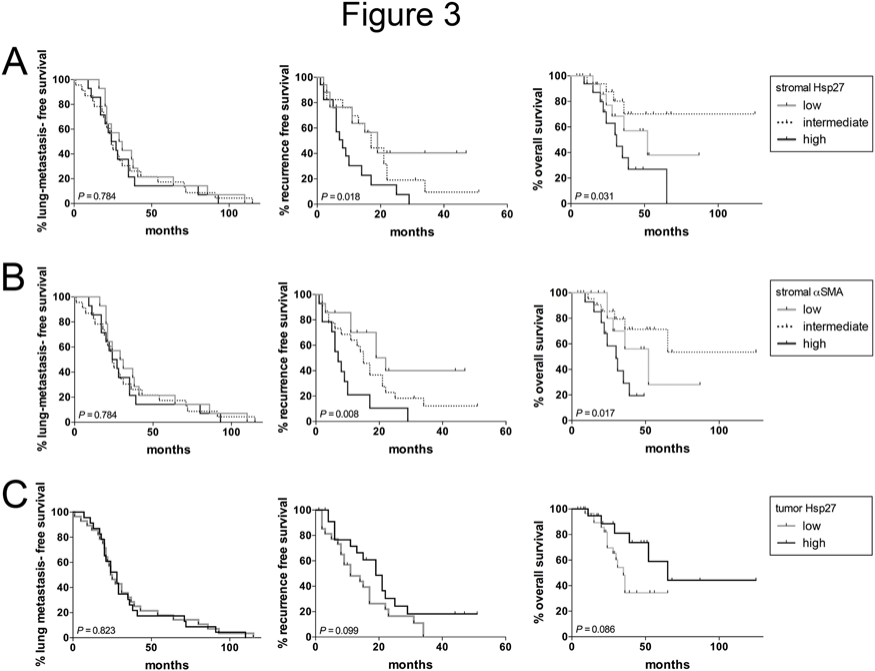
Kaplan-Meier plots showing the lung-metastasis free survival, recurrence free survival and overall survival after metastasectomy dependent on stromal Hsp27 (A), stromal α-SMA (B) and tumor Hsp27 (C) scoring.

We assessed the association of relevant clinicopathological variables with LMFS, RFS and OS after metastasectomy (Table 2). No variable had significant impact on the LMFS. Female patients had a strong trend towards a shorter time to PMs (*P =* 0.091). The stromal expression of neither α-SMA nor Hsp27 was associated with a significantly decreased LMFS. However, both histological markers had a significant prognostic impact on the RFS after pulmonary metastasectomy (*P =* 0.018 and *P =* 0.008 for stromal Hsp27 and α-SMA, respectively) (Fig. 3). Again, female patients showed a strong trend towards decreased RFS. Moreover, patients with a history of previous liver metastases had a decreased RFS compared to patients without liver metastases in the history. However, both variables, sex and the history of previous liver metastases, had no significant influence on the RFS. Additionally to that, extensive stromal Hsp27 stromal Hsp27 and α-SMA were associated with a decreased overall survival after metastasectomy (*P =* 0.031 and *P =* 0.017 for stromal Hsp27 and α-SMA, respectively). Due to the high rate of concordance, stromal Hsp27 and α-SMA were no independent factors in the outcome analysis when adding both variables into a multivariate analysis (data not shown).

**Table 2:** Univariate analysis assessing clinicopathological variables and lung metastasis free survival, recurrence free survival after metastasectomy and overall survival of patients (N = 51) with CRC metastasizing to the lung.

				Lung metastasis free survival			Recurrence free survival			Overall survival		
		Total		Univariate analysis (log-rank)			Univariate analysis (log-rank)			Univariate analysis (log-rank)		
		N = 51	%	Months	HR (95% CI)	p-value	Months	HR (95% CI)	p-value	Months	HR (95% CI)	p-value
**Sex**	**Male**	29	43.1	24	1	0.091	17	1	0.125	39	1	0.464
	**Female**	22	56.9	29	0.62 (0.35–1.09)		11	1.63 (ß.86–3.09)		52	0.72 (0.29–1.77)	
**Age (years)**	**< 64 yrs**	26	51.0	24	1	0.759	15	1	0.820	39	1	0.377
	**≥ 64 yrs**	25	49.0	28	0.92 (0.52–1.61)		11	0.93 (0.49–1.77)		65	0.67 (0.28–1.64)	
**Location**	**Colon**	27	52.9	28	1	0.926	15	1	0.730	52	1	0.671
	**Rectum**	24	47.1	23	1.03 (0.58–1.81)		11	0.90 (0.47–1.70)		36	1.21 (0.50–2.93)	
**T stage**	**pT1+pT2**	8	16.7	24	1	0.932	15	1	0.977	31	1	0.650
	**pT3+pT4**	40	83.3	25	1.03 (0.48–2.24)		14	0.99 (0.41–2.37)		52	0.78 (0.26–2.35)	
	**unknown**	3	-									
**N stage**	**pN0**	21	43.7	29	1	0.643	17	1	0.465	39	1	0.827
	**pN1+pN2**	27	56.3	24	1.15 (0.64–2.07)		11	1.28 (0.65–2.50)		52	0.91 (0.37–2.23)	
	**unknown**	3	-									
**Previous liver metastasis**	**Yes**	16	31.4	25	1	0.691	9	1	0.118	30	1	0.401
	**No**	35	68.6	24	0.89 (0.48–1.62)		17	0.60 (0.31–1.16)		52	0.69 (0.28–1.69)	
**stromal Hsp27**	**low**	17	33.3	25	1		19	1	0.018^a^	52	1	0.031^a^
	**intermediate**	17	33.3	28	0.80 (0.40–1.59)	0.575^a^	17	1.50 (0.68–3.48)		NR	0.48 (0.13–1.70)	
	**high**	17	33.3	24	1.15 (0.58–2.26)		10	2.65 (1.31–6.77)		31	1.98 (0.75–5.37)	
**stromal alpha-SMA**	**low**	14	27.5	29	1		22	1	0.008^a^	52	1	
	**intermediate**	23	45.0	24	1.18 (0.60–2.30)	0.784^a^	15	1.83 (0.77–4.30)		NR	0.58 (0.17–1.90)	0.017^a^
	**high**	14	27.5	24	1.29 (0.61–2.72)		7	3.99 (1.53–10.41)		30	2.57 (0.84–7.84)	
**No. of nodules**	**1**	33	64.7	25	1	0.278	15	1	0.490	52	1	0.653
	**>1**	18	35.3	24	1.38 (0.76–2.50 =		14	1.26 (0.65–2.43)		36	1.23 (0.50–3.03)	
**LMFS**	**<36**	36	70.6	-	-	-	15	1	0.948	39	1	0.512
	**>36**	15	29.4		-		11	1.02 (0.51–2.07)		NR	0.72 (0.26–1.98)	

### Soluble Hsp27 is systemically increased in patients before pulmonary metastasectomy

**Fig 4:**
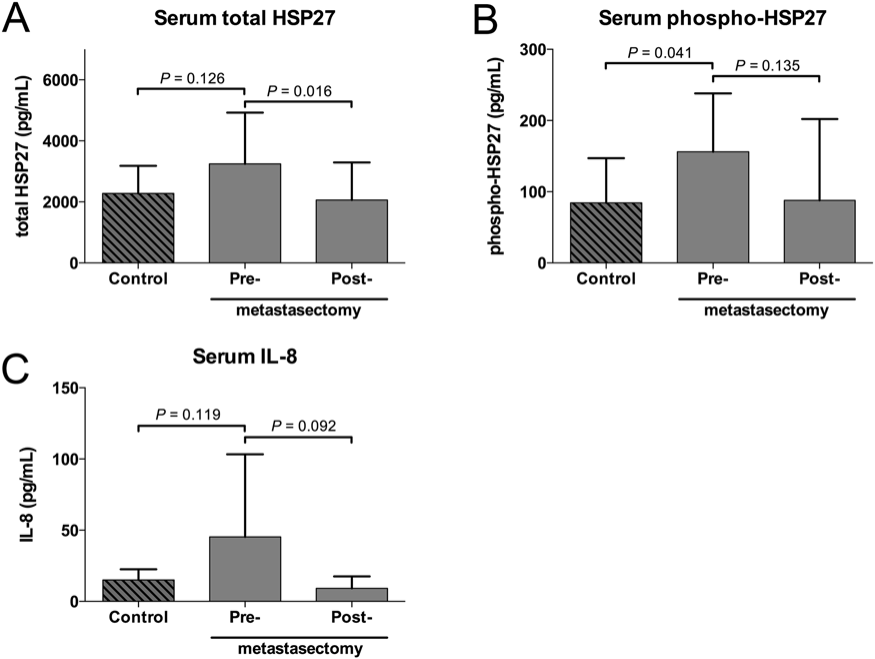
Total Hsp27 (A), phospho-Hsp27 (B) and IL-8 (C) were measured in serum samples of healthy volunteers, patients with CRC lung metastases before and 3 months after metastasectomy (each n = 10; whiskers indicate standard deviation).

Elevated levels of soluble Hsp27 can be detected systemically in various malignant and non-malignant diseases. In a subset of 10 of our patients paired serum samples (pre- and post-metastasectomy) were available (Fig. 4). We compared the serum level of total Hsp27 and phospho-Hsp27 to matched healthy volunteers (S3 Table). Serum total Hsp27 levels were 2276±905, 3245±1684 and 2064±1226 pg/mL (mean±SD) for control, pre- and post-metastasectomy samples, respectively. Total Hsp27 decreased significantly after metastasectomy (*P* = 0.016). Serum phospho-Hsp27 levels were 84±63, 156±82 and 88±114 pg/mL (mean±SD) for control, pre- and post-metastasectomy samples, respectively. A significant difference was found between healthy controls and pre-metastasectomy samples (*P* = 0.041). Although the levels dropped after metastasectomy, the difference was not significant. IL-8, a proangiogenic cytokine involved in the Hsp27 signaling, was detected at very low concentrations of 15±8, 45±58 and 9±8 pg/mL (mean±SD) in the serum samples. The differences of systemic IL-8 levels between the three groups did not differ significantly from each other. No correlation was found between the expression level of stromal Hsp27, tumor Hsp27 and systemic levels (data not shown). Moreover, no significant correlation between total or phospho-Hsp27 and standard inflammatory markers (CRP and fibrinogen) was found (S1B and S1C Fig).

### Stromal Hsp27 expression in pulmonary metastases is significantly increased compared to liver metastases

We further evaluated the stromal expression of stromal Hsp27 and α-SMA in liver metastases of 25 patients. Regarding the stromal Hsp27 expression 0 (0%), 12 (48.0%), 11 (44.0%) and 2 (8.0%) cases were scored as 0, 1+, 2+ and 3+, respectively. Stromal α-SMA expression was rated as expression 0 (0%), 8 (32.0%), 11 (44%) and 6 (24.0%) cases were scored as 0, 1+, 2+ and 3+, respectively. We found a strong correlation between stromal Hsp27 and α-SMA (P < 0.001). Furthermore, stromal Hsp27 positivity was significantly more often found in pulmonary metastases compared to liver metastases (P = 0.014), whereas this difference was not significant for α-SMA (P = 0.625). Moreover, expression of Hsp27 in tumor cells (median 120 (range 0–270)) was significantly increased in liver metastases compared to pulmonary metastases (P = 0.037). We further analyzed the overall survival of the patients with liver metastases depending on their stromal Hsp27, α-SMA and tumor Hsp27 expression, but significant differences were not found (S3 Fig).

## Discussion

Distant metastases are the main cause of cancer-related mortality. Today, the tumor microenvironment and especially fibroblasts become of growing interest, providing additional information on tumor behavior and potential therapeutic targets [32]. Myofibroblasts can be found in the microenvironment of various tumors and extensive fibroblast-activation could be linked to rapid disease progression in malignant disease [32].

Herein we could demonstrate in a well-defined study cohort that vimentin^+^αSMA^+^ stromal cells are highly present in pulmonary metastases of primary CRC. Wikberg *et al*. outlined that myofibroblasts in the tumor center are a better prognosticator for poor prognosis than fibroblasts at the tumor margin [33]. We, therefore, restricted the scoring of the putative fibroblast activation to the central portion of the tumor. Compared to Henry *et al*., who identified myofibroblasts by FAP-staining in primary colon cancer, we found a similar distribution of the stromal scoring (0%, 20%, 38% and 35% for negative, low, intermediate and strong staining, respectively). Additionally, they described the association of high amounts of activated fibroblasts with a decreased overall survival in patients with colon cancer, especially in the metastasized situation [10]. This goes in line with our findings showing a decreased RFS after pulmonary metastasectomy.

Interestingly, α-SMA positive phenotype of tumor-associated stroma was accompanied by the expression of Hsp27. On the one hand, this small heat-shock protein has been examined in CRC tumor cells themselves [34–36]. An increased expression of Hsp27 in tumor cells of primary CRC was described as negative prognosticator for survival [37, 38]. On the other hand, Hsp27 expressing fibroblasts were examined in the context of wound healing and fibrosis. To the best of our knowledge, stromal Hsp27 has not been described as a potential prognostic marker in CRC. Interestingly, strong stromal Hsp27 expression was associated with high MVD in the primary tumors (P = 0.031) and in PM (P = 0.081). Due to the strong correlation of Hsp27 with α-SMA as an established marker for fibroblast activation, the over-expression of Hsp27 was associated with significantly worse clinical outcome after pulmonary metastasectomy (median RFS 19, 17 and 8 months for 1+, 2+ and 3+, respectively), comparable to the prognostic impact of stromal α-SMA expression. The decrease RFS was also translated in a significantly different OS between the low, intermediate and high stromal Hsp27 or α-SMA, respectively (Fig. 3). Similarly, Tsujino *et al*. described α-SMA positive fibroblasts as being capable to predict disease recurrence in a cohort of patients with stage II and III primary CRC [12]. Kahlert *et al*. demonstrated by microdissection of primary CRC, lung and liver metastases that a panel of pro-angiogenic factors are differentially expressed in tumor cells and the stromal compartment. The stromal expression of angiopoietin-2 in pulmonary metastases was an independent prognosticator for poor survival after surgery in multivariate analysis (*P* = 0.044), even in a rather small study cohort (n = 25) [39]. These findings support the results of our work, in which we could describe Hsp27 as a further proangiogenic, stromal marker with prognostic impact after metastasectomy. It is also important to mention, that Sato *et al*. described high stromal expression of the description factors ETS1 as a predictor of CRC lung metastases [40]. Interestingly, ETS1 is strongly interacting with the small heat-shock protein network [41].

The analysis of blood samples in a subgroup of patients revealed that metastatic CRC is a relevant inducer even of systemic Hsp27 expressions. The level of soluble total Hsp27 and phospho-Hsp27, a polymerized form of Hsp27, significantly decreased after complete removal of the PMs. Moreover, compared to a matched control group of healthy volunteers, pre-operative total Hsp27 and phospho-Hsp27 levels were increased and dropped after surgery to comparable low levels. Similar to our findings, Zhao *et al*. could demonstrate increased serum levels of Hsp27 in ovarian cancer with peritoneal metastases. In the same work it was also shown that the levels dropped after chemotherapy [42]. IL-8, which is thought to mediate locally the proangiogenic effect of Hsp27, was also elevated before metastasectomy without reaching significance [22]. Nontheless, the circulating levels of Hsp27 did not correlate with the stromal or tumor expression. Furthermore it did not correlate with the systemic levels of CRP or fibrinogen. Thus, the circulating Hsp27 might be influenced by other factors like secretion, degradation and elimination. Based on these preliminary results, further studies with adequate cohort sizes will be necessary to further clarify the possible prognostic and predictive role of circulating Hsp27 levels in patients with CRC.

In patients with CRC liver metastases the tumor stroma Hsp27^+^ vimentin^+^αSMA^+^ fibroblasts were less evident than in pulmonary metastases. In contrast to this, the Hsp27 expression in tumor cells was significantly higher in resected liver metastases compared to pulmonary metastases. This goes in line with the observation that the p38 MAPK signaling, a pivotal kinase for Hsp27 phosphorylation and activation, is downregulated in CRC pulmonary metastases compared to liver metastases [43].

The findings of this work are of translational relevance because of two aspects: first, Hsp27, systemically, or confined to the tumor stroma, might possess potential prognostic and predictive value. Pulmonary metastasectomy is a widely offered treatment option in patients with CRC PM. However, the identification of patients who will benefit from surgery alone or an optional adjuvant chemotherapy is a matter of current research [44]. Biomarkers like Hsp27 might help to identify patients with high risk of early recurrence of disease after surgery. Adjuvant chemotherapy and a stringent follow-up could be offered to these patients. During the follow-up of patients with malignant disease, serum markers, e.g. CEA, CA19–9 and beta-HCG, are routinely used nowadays and additional markers might potentiate the accuracy of these established markers. A limitation to this study is, that CEA and CA19–9 levels were not routinely determined before metastasectomy. Correlation of these established tumor markers with serum Hsp27 will be addressed in a future study.

Secondly, Hsp27 is a promising drug target. Apatorsen (OGX-427, Oncogenex, Bothell, Washington, USA), a modified antisense oligonucleotide, binds to the Hsp27 mRNA transcript and therefore inhibits the Hsp27 expression [45]. Currently ongoing Phase II studies are recruiting patients with advanced prostate, bladder, pancreatic and non-small cell lung cancer [46]. The elevated expression levels of Hsp27 in the tumor-associated stroma provide a rationale for the use of Hsp27 also in patients with primary CRC. Given that not only CRC lung metastases, but also liver and lymph node metastases exhibit strong myofibroblast recruitment, especially patients with metastatic disease might benefit from Hsp27 inhibition.

In conclusion, we could demonstrate that Hsp27 is co-expressed with αSMA in the tumor stroma of CRC lung metastases and, moreover, that its over-expression is associated with worse clinical outcome after metastasectomy. Of note, soluble Hsp27 was also systemically measurable, making serum Hsp27 a potential future serum marker in CRC.

## Supporting Information

S1 Table Antibodies and dilutions used for immunohistochemistry and immunofluorescence. (DOCX)

S2 Table Descriptive data on patient, primary tumor and metastases characteristics stratified by MVD and vimentin (N = 51). (DOCX)

S3 Table Characteristics of matched patients and healthy controls. (DOCX)

S1 Fig Hsp27 expression was also found in tumor cells itself (anti-Hsp27; DAB substrate; 200x magnification) (A). Correlation of pre-operative Hsp27 and C-reactive protein (CRP) or fibrinogen (n = 10) (B and C). Neither total Hsp27 (B), nor phospho-Hsp27 (C) correlated significantly with CRP or fibrinogen.
(TIF)

S2 Fig In CRC liver metastases stromal Hsp27 and α-SMA scoring correlated significantly (n = 25) (A). (TIF)

S3 Fig The distribution of stromal Hsp27 (A) differed significantly in liver metastases (n = 25) compared to lung metasatses (n = 51). This difference did not reach significance for stromal α-SMA (B). Hsp27 expression in tumor cells was significantly higher in liver metastases compared to lung metastases (C). No significant differences were observed between the subgroups regarding overall survival (D-F).
(TIF)
