# Supplementary material for: Stromal Expression of Heat-Shock Protein 27 Is Associated with Worse Clinical Outcome in Patients with Colorectal Cancer Lung Metastases
Source: PLoS One. 2015 Mar 20;10(3):e0120724. doi: 10.1371/journal.pone.0120724 (PMC4368667; doi:10.1371/journal.pone.0120724)
Supplement: S1 Table — (DOCX) [file pone.0120724.s001.docx]

**Supplementary Table 1**

| **Antibody** | **Manufacturer** | **Application** | **Dilution** | **Antigen retrieval** |
| --- | --- | --- | --- | --- |
| Rabbit polyclonal alpha-SMA (ab5694) | Abcam; Cambridge, UK | IF/IHC | 1:300/1:600 | heat-mediated, buffer EDTA pH=9 |
| Alexa Fluor® 488 Goat Anti-Rabbit IgG (A-11008) | Molecular probes, Carlsbad, CA, USA | IF | 1:500 | - |
| ImmPRESS Reagent Kit Anti-Rabbit IgG (MP-7401) | Vector Laboratories, Burlingame, CA, USA | IHC | prediluted | - |
| Mouse monoclonal CD31 (JC70A) | Dako, Carpenteria, CA, USA | IHC | 1:20 | heat-mediated, buffer EDTA pH=9 |
| ImmPRESS Reagent Kit Anti-Mouse IgG (MP-7402) | Vector Laboratories, Burlingame, CA, USA | IHC | prediluted | - |
| Mouse monoclonal Hsp27 (sc-131132) | Santa Cruz Biotechnology, Santa Cruz, CA, USA | IF/IHC | 1:400 | heat-mediated, buffer EDTA pH=8 |
| Alexa Fluor® 546 Goat Anti-Mouse IgG (H+L) (A-11003) | Molecular probes, Carlsbad, CA, USA | IF | 1:500 | - |
| ImmPRESS Reagent Kit Anti-Mouse IgG (MP-7402) | Vector Laboratories, Burlingame, CA, USA | IHC | prediluted | - |
| Mouse monoclonal Vimentin (#M0725) | Dako, Carpenteria, CA, USA | IHC | 1:300 | heat-mediated, Target Retrieval Solution (Dako, Carpenteria, CA, USA) |
| UltraVision LP detection system | Lab Vision Corporation, Fremont, CA, USA | IHC | prediluted |  |
