# Supplementary material for: Stromal Expression of Heat-Shock Protein 27 Is Associated with Worse Clinical Outcome in Patients with Colorectal Cancer Lung Metastases
Source: PLoS One. 2015 Mar 20;10(3):e0120724. doi: 10.1371/journal.pone.0120724 (PMC4368667; doi:10.1371/journal.pone.0120724)
Supplement: S2 Table — (DOCX) [file pone.0120724.s002.docx]

**Supplementary Table 3**

|  |  |  | | **MVD** | | | | | **Vimentin** | | | | | | |
| --- | --- | --- | --- | --- | --- | --- | --- | --- | --- | --- | --- | --- | --- | --- | --- |
|  |  | **Total** | | **low** | | **high** | | **Χ^2^** | **low** | | **intermediate** | | **high** | | **Χ^2^** |
|  |  | **N=51** | **%** | **N=25** | **%** | **N=26** | **%** | **p-value** | **N=4** | **%** | **N=21** | **%** | **N=26** | **%** | **p-value** |
| **Patients** | |  |  |  |  |  |  |  |  |  |  |  |  |  |  |
| **Sex** | **Male** | 29 | 56.9 | 16 | 31.4 | 13 | 25.5 | 0.313 | 2 | 3.9 | 13 | 25.5 | 14 | 27.5 | 0.913 |
|  | **Female** | 22 | 43.1 | 9 | 17.6 | 13 | 25.5 |  | 2 | 3.9 | 8 | 15.7 | 12 | 23.5 |  |
| **Age (years)** | **Median** | 63 | | 67 | | 58.5 | | 0.048 | 63.5 | | 67 | | 57.5 | | 0.180^a^ |
|  | **Range** | 33-83 | | 33-83 | | 45-77 | |  | 50-68 | | 44-83 | | 33-78 | |  |
| **Primary tumor** | |  |  |  |  |  |  |  |  |  |  |  |  |  |  |
| **Location** | **Colon** | 27 | 52.9 | 14 | 27.5 | 13 | 25.5 | 0.668 | 1 | 2.0 | 12 | 23.5 | 14 | 27.5 | 0.585 |
|  | **Rectum** | 24 | 47.1 | 11 | 21.5 | 13 | 25.5 |  | 3 | 5.9 | 9 | 17.6 | 12 | 23.5 |  |
| **T stage** | **pT1** | 1 | 2.1 | 0 | 0.0 | 1 | 2.1 |  | 0 | 0.0 | 1 | 2.1 | 0 | 0.0 |  |
|  | **pT2** | 7 | 14.6 | 3 | 6.2 | 4 | 8.3 | 0.848 | 0 | 0.0 | 3 | 6.2 | 4 | 8.3 | 0.186 |
|  | **pT3** | 34 | 70.8 | 19 | 39.6 | 15 | 31.2 |  | 4 | 8.3 | 10 | 20.8 | 20 | 41.7 |  |
|  | **pT4** | 6 | 12.5 | 3 | 6.2 | 3 | 6.2 |  | 0 | 0.0 | 5 | 10.4 | 1 | 2.1 |  |
|  | **N/A** | 3 | - |  |  |  |  |  |  |  |  |  |  |  |  |
| **N stage** | **pN0** | 21 | 43.8 | 12 | 25.0 | 9 | 18.8 | 0.709 | 1 | 2.1 | 9 | 18.8 | 11 | 22.9 | 0.456 |
|  | **pN1** | 11 | 22.9 | 6 | 12.5 | 5 | 10.4 |  | 0 | 0.0 | 5 | 10.4 | 6 | 12.5 |  |
|  | **pN2** | 16 | 33.3 | 7 | 14.6 | 9 | 18.8 |  | 3 | 8.3 | 5 | 10.4 | 8 | 16.7 |  |
|  | **N/A** | 3 | - |  |  |  |  |  |  |  |  |  |  |  |  |
| **Grading** | **G1** | 2 | 3.9 | 2 | 3.0 | 0 | 0.0 |  | 1 | 2.0 | 0 | 0.0 | 1 | 2.0 |  |
|  | **G2** | 42 | 82.4 | 19 | 37.3 | 23 | 45.1 | 0.319 | 2 | 2.9 | 18 | 35.3 | 22 | 43.1 | 0.218 |
|  | **G3** | 7 | 13.7 | 4 | 7.8 | 3 | 5.9 |  | 1 | 2.0 | 3 | 5.9 | 3 | 5.9 |  |
| **Pulmonary metastasis** | |  |  |  |  |  |  |  |  |  |  |  |  |  |  |
| **Previous liver metastasis** | **Yes** | 16 | 31.4 | 8 | 15.7 | 8 | 15.7 | 0.925 | 0 | 0.0 | 7 | 13.7 | 9 | 17.6 | 0.442 |
|  | **No** | 35 | 68.6 | 17 | 33.3 | 18 | 35.3 |  | 4 | 7.8 | 14 | 27.5 | 17 | 33.3 |  |
| **No. of nodules** | **1** | 33 | 64.7 | 16 | 31.4 | 17 | 33.3 | 0.918 | 2 | 3.9 | 11 | 21.6 | 20 | 39.2 | 0.195 |
|  | **>1** | 18 | 35.3 | 9 | 17.6 | 9 | 17.6 |  | 2 | 3.9 | 10 | 19.6 | 6 | 11.8 |  |
| **Stromal HSP27** | **low** | 17 | 33.3 | 10 | 19.6 | 7 | 13.7 |  | 3 | 5.9 | 12 | 23.5 | 2 | 3.9 |  |
|  | **intermediate** | 17 | 33.3 | 8 | 15.7 | 9 | 17.6 | 0.577 | 1 | 2.0 | 8 | 15.7 | 8 | 15.7 | <0.001 |
|  | **high** | 17 | 33.3 | 7 | 13.7 | 10 | 19.6 |  | 0 | 0.0 | 1 | 2.0 | 16 | 31.4 |  |
| **Stromal alpha-SMA** | **low** | 14 | 27.5 | 9 | 17.6 | 5 | 9.8 |  | 4 | 7.8 | 10 | 19.6 | 0 | 0.0 |  |
|  | **intermediate** | 23 | 45.1 | 11 | 21.6 | 12 | 23.5 | 0.315 | 0 | 0.0 | 11 | 21.6 | 12 | 23.5 | <0.001 |
|  | **high** | 14 | 27.5 | 5 | 9.8 | 9 | 17.6 |  | 0 | 0.0 | 0 | 0.0 | 14 | 27.5 |  |

^a^Kruskal-Wallis test
