# Supplementary material for: Stromal Expression of Heat-Shock Protein 27 Is Associated with Worse Clinical Outcome in Patients with Colorectal Cancer Lung Metastases
Source: PLoS One. 2015 Mar 20;10(3):e0120724. doi: 10.1371/journal.pone.0120724 (PMC4368667; doi:10.1371/journal.pone.0120724)
Supplement: S3 Table — (DOCX) [file pone.0120724.s003.docx]

**Supplementary Table 2**

|  | **healthy controls**  **n= 10** | **CRC patients**  **n= 10** | **p-value** |
| --- | --- | --- | --- |
| **Sex**  **male/female** | 7/3 | 7/3 | *p=* 1.000^a^ |
| **Age**  **range (median)** | 45-88 (52.5) | 44-76 (62.5) | *p=* 0.430^b^ |
| **Smoking history**  **never smoker/ever smoker** | 4/6 | 3/7 | *p=* 1.000^a^ |
| **Months after primary tumor resection (range)** | - | 29 (7-91) | *-* |

^a^Chi square test; ^b^Mann-Whitney test
