# Supplementary figures and images for: Stromal Expression of Heat-Shock Protein 27 Is Associated with Worse Clinical Outcome in Patients with Colorectal Cancer Lung Metastases
Source: PLoS One. 2015 Mar 20;10(3):e0120724. doi: 10.1371/journal.pone.0120724 (PMC4368667; doi:10.1371/journal.pone.0120724)

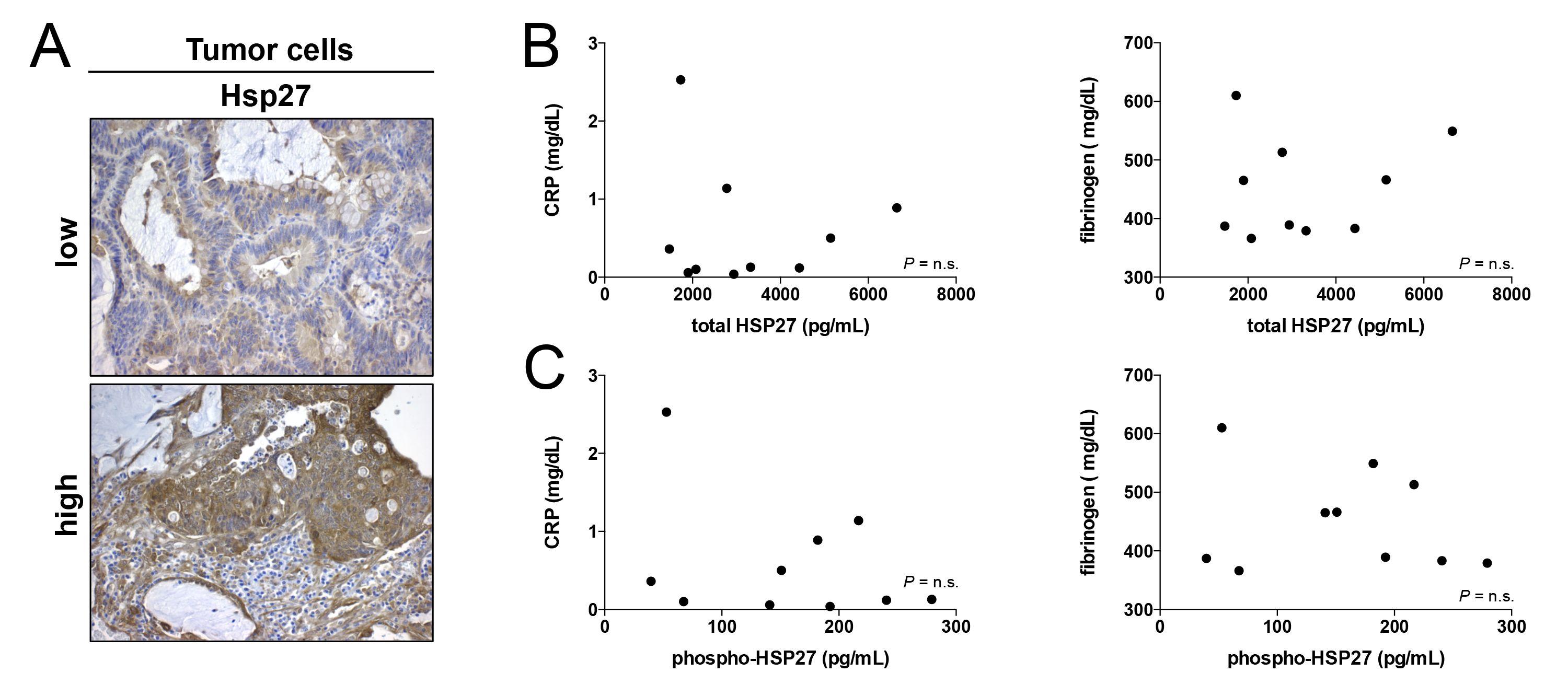

Supplement: S1 Fig — Correlation of pre-operative Hsp27 and C-reactive protein (CRP) or fibrinogen (n = 10) (B and C). Neither total Hsp27 (B), nor phospho-Hsp27 (C) correlated significantly with CRP or fibrinogen. (TIF) [file pone.0120724.s004.tif]

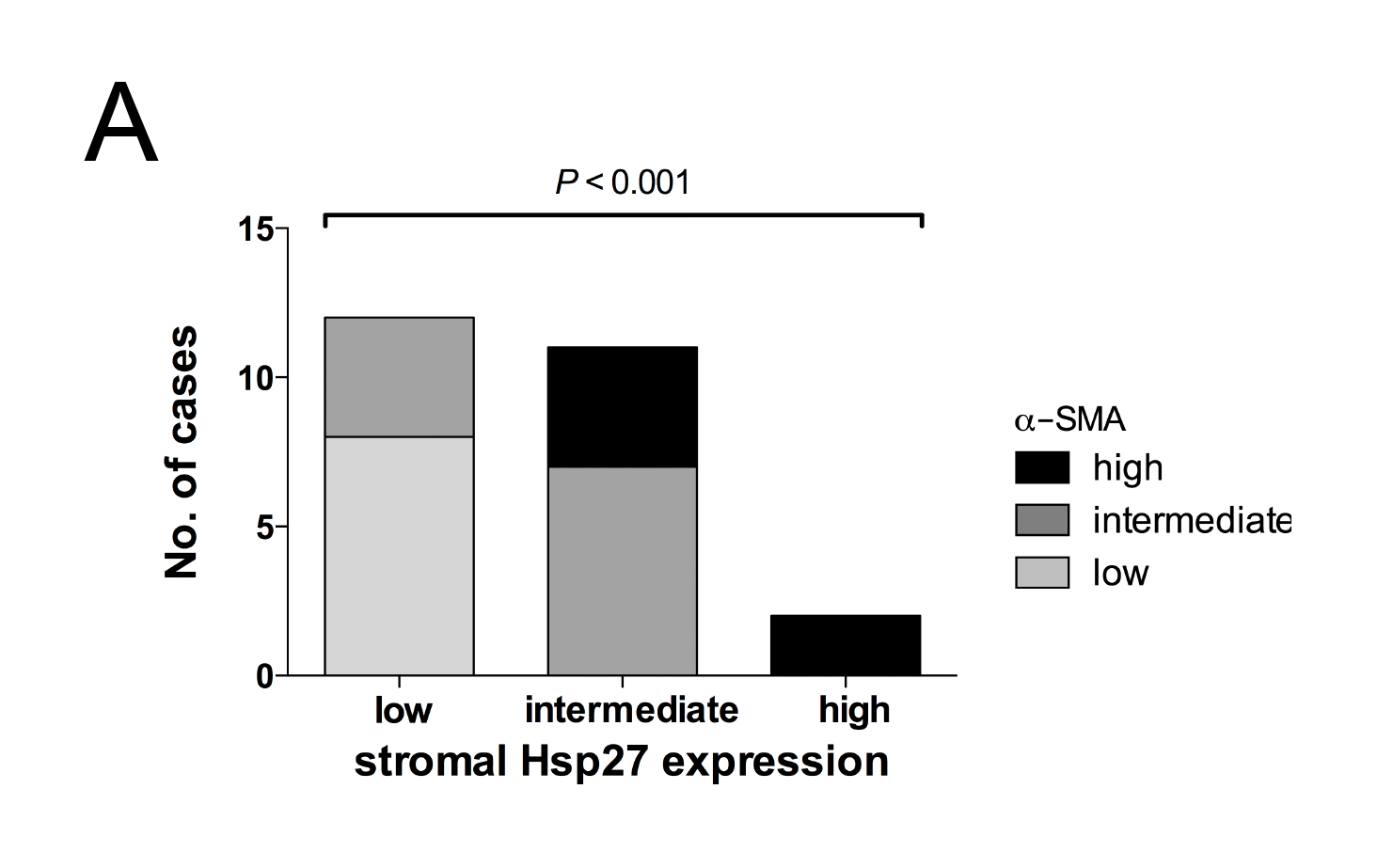

Supplement: S2 Fig — (TIF) [file pone.0120724.s005.tif]

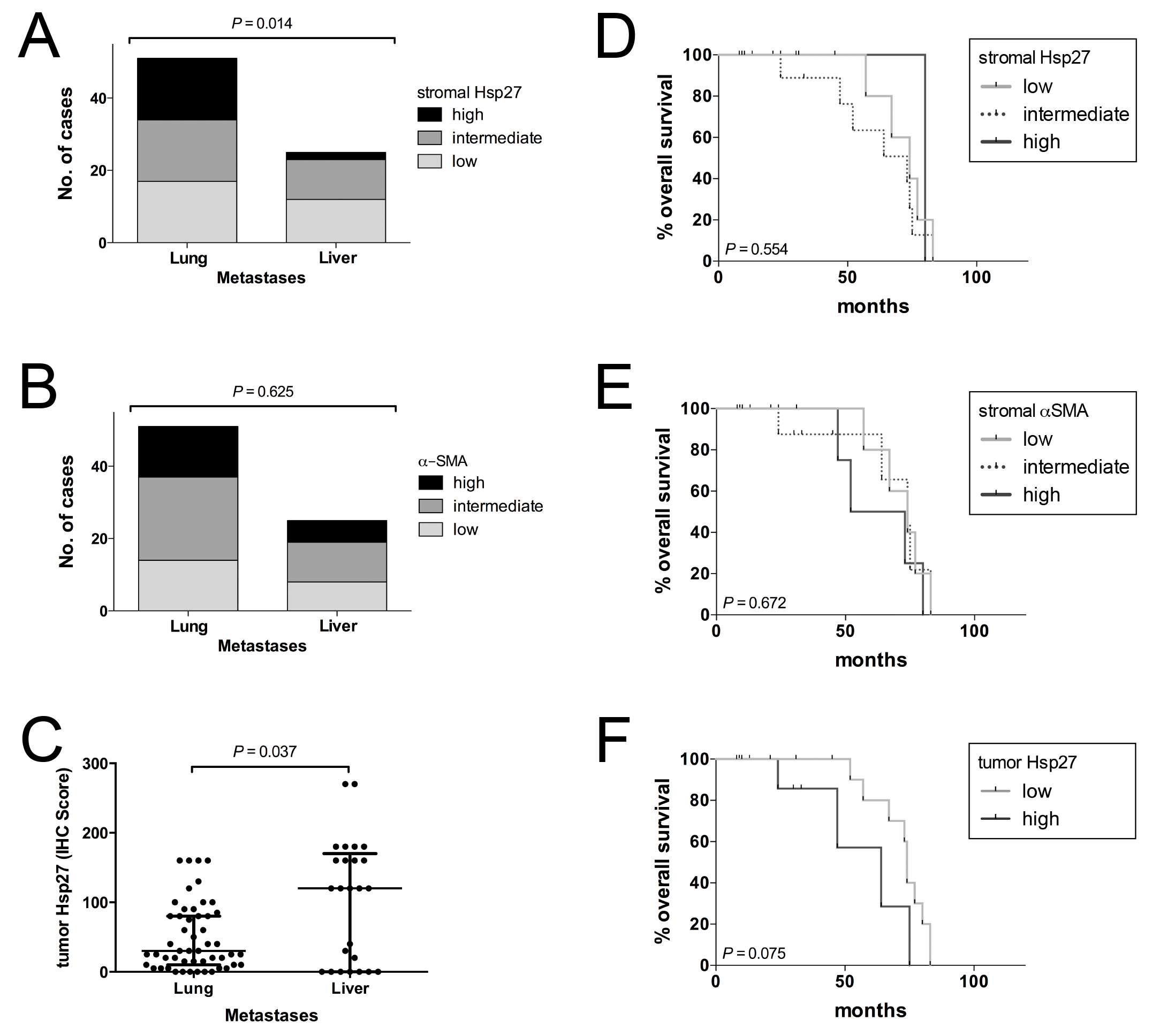

Supplement: S3 Fig — This difference did not reach significance for stromal α-SMA (B). Hsp27 expression in tumor cells was significantly higher in liver metastases compared to lung metastases (C). No significant differences were observed between the subgroups regarding overall survival (D-F). (TIF) [file pone.0120724.s006.tif]
